# Differences in stem cell marker and osteopontin expression in primary and recurrent glioblastoma

**DOI:** 10.1186/s12935-022-02510-4

**Published:** 2022-02-19

**Authors:** Bülent Polat, Gisela Wohlleben, Rebekka Kosmala, Dominik Lisowski, Frederick Mantel, Victor Lewitzki, Mario Löhr, Robert Blum, Petra Herud, Michael Flentje, Camelia-Maria Monoranu

**Affiliations:** 1 Department of Radiation Oncology, University of Würzburg, Würzburg, Germany; 2 Department of Neurosurgery, University of Würzburg, Würzburg, Germany; 3 Department of Neurology, University Hospital Würzburg, Würzburg, Germany; 4 Department of Neuropathology, Institute of Pathology, University of Würzburg, Würzburg, Germany; 5 Department of Radiation Oncology, University Hospital Würzburg, Josef-Schneider-Str. 11, 97080 Würzburg, Germany

**Keywords:** Glioblastoma, Glioma stem cells, Osteopontin, CD133, Nestin

## Abstract

**Background:**

Despite of a multimodal approach, recurrences can hardly be prevented in glioblastoma. This may be in part due to so called glioma stem cells. However, there is no established marker to identify these stem cells.

**Methods:**

Paired samples from glioma patients were analyzed by immunohistochemistry for expression of the following stem cell markers: CD133, Musashi, Nanog, Nestin, octamer-binding transcription factor 4 (Oct4), and sex determining region Y-box 2 (Sox2). In addition, the expression of osteopontin (OPN) was investigated. The relative number of positively stained cells was determined. By means of Kaplan–Meier analysis, a possible association with overall survival by marker expression was investigated.

**Results:**

Sixty tissue samples from 30 patients (17 male, 13 female) were available for analysis. For Nestin, Musashi and OPN a significant increase was seen. There was also an increase (not significant) for CD133 and Oct4. Patients with mutated Isocitrate Dehydrogenase-1/2 (IDH-1/2) status had a reduced expression for CD133 and Nestin in their recurrent tumors. Significant correlations were seen for CD133 and Nanog between OPN in the primary and recurrent tumor and between CD133 and Nestin in recurrent tumors. By confocal imaging we could demonstrate a co-expression of CD133 and Nestin within recurrent glioma cells. Patients with high CD133 expression had a worse prognosis (22.6 vs 41.1 months, p = 0.013). A similar trend was seen for elevated Nestin levels (24.9 vs 41.1 months, p = 0.08).

**Conclusions:**

Most of the evaluated markers showed an increased expression in their recurrent tumor. CD133 and Nestin were associated with survival and are candidate markers for further clinical investigation.

**Supplementary Information:**

The online version contains supplementary material available at 10.1186/s12935-022-02510-4.

## Background

Glioblastoma (GB) is the most common, rapidly growing and very aggressive primary brain tumor in adults [1]. The standard treatment approach with surgery followed by adjuvant radio-chemotherapy is not successful in eliminating all tumor cells permanently and has only a limited curative effect with a median overall survival less than 2 years [2–4]. Recurrence usually occurs within the multimodal treatment. One reason for treatment failure seems to be the presence of so-called cancer stem-like cells (CSCs), a subpopulation of tumor cells, which persist in the patient [5]. Like embryonal stem cells (ESCs), these highly malignant cells are characterized by perpetual self-renewal, proliferation and differentiation. CSCs are responsible for tumor initiation, its invasive growth and metastasis formation and therefore influence tumor formation and progression. They are resistant to radio- and chemotherapy, can survive in hypoxic tissue areas and reside within perivascular niches [6, 7]. CSCs can behave silent for a long period of time and play an important role in tumor recurrence [8]. The problem is still the identification of these stem cells within the tumor. Some characteristic markers are enriched in glioblastoma but there is no single one or a combination of markers which are recommended for diagnosis. A reliable identification of CSCs would therefore be very helpful for future targeted therapies. In this work putative stem cell markers for GB, CD133, Nestin, Musashi and the transcription factors Sox2, Oct4 and Nanog are investigated.

The membrane bound glycoprotein CD133, also called prominin-1, is a human neural stem cell marker. As a cell surface marker of CSCs, it seems to play a key role in tumor invasion and recurrence [9]. Increase in CD133 expression positively correlates with radio-resistance leading to relapse, increase of malignancy and poor survival [6]. Nestin is a member of the intermediate filament protein family and necessary during neural development. In adults it is expressed in neural progenitor cells and in several cancer types, including glioma [10]. An association between increased Nestin expression and gliomas of a higher grade could be shown. Its expression was correlated with increased malignancy, invasiveness and tumor cell dedifferentiation [11, 12]. Nestin knockdown caused suppression of invasion, migration, and proliferation of human GB cell lines. In return, increased F-actin expression and cell adhesion was observed [13]. However, the value of Nestin as prognostic marker is controversially discussed [14, 15]. Another CSC-related marker is the evolutionary highly conserved RNA-binding protein Musashi, which is not only important for neural and glial development, but also for tumorigenesis [16]. This protein seems to be involved in cell proliferation, differentiation, apoptosis, cell cycle regulation, as well as self-renewal and maintenance of pluripotency of stem cells [17, 18]. Normally expressed in progenitor cells, high levels of Musashi were also detected in GB and many tumors of different entities. The prevention of apoptosis and the induction of proliferation seems to be related to Musashi [19, 20]. Recently it has been shown that Musashi acts as a “key oncogenic factor of GB” and contributes to invasion as well as radio-resistance and tumor recurrence [21]. The nuclear transcription factor Sox2 is expressed during mammalian embryogenesis in neural progenitor cells and is overexpressed in a variety of tumors, including all grades of gliomas [22]. In glioma the rate of Sox2 expression correlates positively with the grade of malignancy [23]. An extraordinary high expression rate of Sox2 is detected in GB with about 80–85% positive tumor cells. In 10–14% of GB an amplification of the Sox2 gene occurs [24]. Two other stem cell associated transcription factors and key regulators of pluripotency are Oct4 and Nanog, both share many of their target genes with Sox2 [25]. Together with Sox2 they are expressed in nearly all gliomas and their expression rate increases with tumor grade [26]. Osteopontin, an actively secreted glycoprotein, is overexpressed in many tumors as well as in glioma [27]. It was shown that patients with high serum levels of OPN have worse prognosis [28]. OPN is overexpressed in hypoxia, [29] associated with CSCs in periarteriolar niches and is suggested to promote tumor recurrence by supporting migration of the CSCs out of these niches [30]. Furthermore, OPN plays an essential role for tumorigenicity, maintenance of stemness of CSCs [31] and is involved in DNA damage repair post irradiation promoting radio-resistance of glioma cells [32].

Certain molecular markers in glioblastoma have high prognostic value. The methylation of O^6^-methylguanine DNA methyltransferase (MGMT) and IDH-1/2 mutation are associated with both prolonged progression free survival and overall survival [33, 34]. In addition, MGMT methylation status is predictive for treatment response with temozolomide [2].

Published data also suggest that α-thalassemia/mental retardation syndrome X-linked gene (ATRX) loss is associated with prolonged overall survival (OS) in IDH-1/2 wild-type GB patients [35, 36].

## Materials and methods

### Patients

This study includes 60 archived tumor sections from 30 patients with confirmed primary and recurrent glioma classified according to the World Health Organization guidelines from 2016. All patients underwent resection of the primary tumor followed by standard adjuvant radio-chemotherapy, radiation alone or chemotherapy alone. No patient received neo-adjuvant treatment before primary resection. Molecular markers were determined from untreated primary tumor tissue only. During follow-up, all patients underwent second surgery for tumor recurrence and received further adjuvant or salvage/palliative treatment (Table 1 and Additional file 1: Table S1).

**Table 1:** Patient and treatment characteristics

Characteristics	Value/number (%)
Patient number	30
Gender	
Male	17 (57)
Female	13 (43)
Treatment time	07/2004–04/2017
Age at diagnosis, median (range)	45.8 (23.2–69.4)
< 40	10 (33)
40–< 60	15 (50)
60–< 70	5 (17)
WHO grade	
Primary tumor	Grade II: 1
	Grade III: 1
	Grade IV: 28 (93)
Recurrent tumor	Grade III: 1
	Grade IV: 29 (97)
Molecular markers	
MGMT methylation	13/30 (43)
IDH1/2 mutation	8/30 (27)
ATRX mutation	9/30 (30)
Treatment, 1st line	
Surgery only	2
Surgery + adjuvant radio-chemotherapy	27 (90)
Surgery + adjuvant radiotherapy	1
Treatment, 2nd line	
Surgery only	4 (13)
Surgery + radio-chemotherapy	7 (23)
Surgery + radiotherapy	2 (7)
Surgery + chemotherapy	17 (57)
Median time from first to second surgery (months, range)	8.1 (3.4–72.8)
Median follow-up (months)	24.0
Vital status at last follow up	
Alive	7 (23)
Dead	23 (77)

### Immunohistochemistry (IHC) staining

For histopathological analysis formalin fixed and paraffin embedded (FFPE) samples were assessed and graded using standard haematoxylin and eosin (H&E) sections (3–4 µm). The astrocytic origin was confirmed by glial fibrillary acidic protein (GFAP) staining. Immunohistochemistry for detection of the IDH1 R132H mutation was performed using the monoclonal antibody anti-IDH1 R132H (clone H09, 1:50, Dianova, Hamburg, Germany). For detection of Alpha-thalassemia/mental retardation syndrome X-linked (ATRX) mutation, which result in the loss of nuclear expression, the antibody anti-ATRX (clone AX1, 1:200, Dianova, Hamburg, Germany) was used. In addition, MGMT promoter methylation status was assessed using methylation-specific pyrosequencing. Histological classification, molecular genetic analysis and tumor grading was accomplished by an experienced neuropathologist. To recognize any differences in the expression of the selected stem cell markers between the paired glioma samples, immunohistochemically staining was performed as follows: FFPE sections from the primary and recurrent tumor were deparaffinized in 100% xylene and rehydrated using graded alcohol series (100, 96, 70% for 5 min each). For antigen retrieval the specimens were heat-treated with 20 mM citric acid buffer, pH 6.0, in a pressure cooker for 10 min and cooked in TRS, pH 9, for 5 min for CD133 staining, respectively. Afterwards, endogenous peroxidase activity was inactivated by incubating the sections in 3% H_2_O_2_ for 15 min. Then sections were blocked with 10% normal goat serum (Thermo Fisher Scientific, Waltham, MA, USA) for 20 min at room temperature and then incubated overnight at 4 °C with primary antibodies. The antibodies were directed against the stem cell markers CD133/1 (1:100, Cat# 130-090-422; Miltenyi Biotec, Bergisch Gladbach, Germany), Musashi (1:250, Cat# D270-3; MBL International, Woburn, Massachusetts, USA), Nanog (1:400, Cat# 4903; Cell Signaling, Danvers, Massachusetts, USA), Nestin (1:2400, Cat# MAB1259; R&D-Systems Inc., Minneapolis, USA), Oct 3/4 (1:50, Cat# sc-5279; Santa Cruz Biotechnology Inc., Dallas, Texas, USA), OPN (1:400, Cat# sc-21742; Santa Cruz Biotechnology Inc., Dallas, Texas, USA) and Sox2 (1:300, Cat# MAB2018; R&D-Systems Inc., Minneapolis, USA).

Next day, detection was performed using a multi-link kit (Super Sensitive Link-Label IHC Detection System, BioGenex, Fremont, California, USA), consisting of a link antibody and a label antibody, followed by development in 3, 3′-diaminobenzidine tetrahydrochloride (DAB) solution (Dako, Glostrup, Denmark). The sections were counterstained with hematoxylin for 2 min, dehydrated by inverse graded alcohol series (70, 96 and 100%) and mounted permanently with Histokit II (Roth, Karlsruhe, Germany). The percentage of stained cells per tumor area (100 tumor cells) was determined by standard bright-field microscopy using a 20 × objective. A total of five different areas were examined. Normal brain from autopsies was used as negative control. Appropriate positive control tissues for the primary antibodies were colon carcinoma (CD133), testis (Musashi), seminoma (Nanog and Oct4), kidney (Nestin) and ovarian cancer (OPN and Sox2).

### Immunofluorescence staining and imaging

For detection of a possible co-localization of CD133 and Nestin we established an immunofluorescence approach. For immunofluorescence staining, sections were deparaffinized and rehydrated as described above. Antigen retrieval was performed for 25 min in 20 mM citric acid buffer (pH 6.0), in a pressure cooker. Sections were rinsed with dH_2_0 and in Tris-buffered saline (TBS) and were treated with 10% horse serum, 0.3% Triton X100 in TBS, for 1 h at room temperature. As primary antibodies, anti-human CD133 (Sigma-Aldrich, clone 2F8, ZooMAb^®^ rabbit monoclonal, 1:50) and Nestin (R&D Systems, MAB1259, mouse monoclonal, 1:2400) were used. Antibodies were diluted in antibody-dilution buffer (DCS—Innovative Diagnostik Systeme, Hamburg, Germany) and incubated overnight. Next day, sections were washed twice in TBS and treated for 3 h at room temperature with the corresponding secondary antibodies (Jackson ImmunoResearch Laboratories, Inc., Pennsylvania, USA; stock solutions with 0.55 mg/ml IgG). The following secondary antibodies were used: donkey anti-rabbit IgG affiniPure (H + L)-Cy3-550 (1:400) and goat anti-mouse IgG affiniPure (H + L)-Alexa488 (1:400). Sections were washed twice in 1 × TBS and stained with 4′, 6-diamidino-2-phenylindole (DAPI) (2 mg/ml stock solution, freshly diluted 1:5000 in 1 × TBS) for 5 min at room temperature. Sections were washed twice in 1 × TBS and were finally embedded in Aquapolymount (Polysciences). Kidney tissue was used as positive control for anti-Nestin. For negative controls, biopsy samples from healthy donors (from autopsies) were used. Cross-reactivity of secondary antibodies was tested by using secondary antibodies in the absence of corresponding primary antibodies.

### Confocal laser scanning microscopy and image processing

Confocal imaging was performed with an IX81 Olympus microscope, an Olympus FV1000 confocal laser scanning system with a FVD10 SPD spectral detector and diode lasers (405, 473, 559 and 635 nm laser lines). Images were acquired with an Olympus UAPO 20x (air, numerical aperture 0.75) objective. For confocal scanning, a pinhole setting representing one Airy disc was used. 12-bit z-stack images were processed by maximum intensity projection and were adjusted in brightness and contrast using Image J software (Rasband, W.S., ImageJ, US National Institutes of Health, Bethesda, Maryland, USA, https://imagej.nih.gov/ij/). Images are shown as RGB images (8-bit per color channel). Fluorescence images were processed for final presentation using Adobe Photoshop CS5.

### Statistics

All statistical analyses were done with SPSS for Windows version 25.0 (IBM SPSS, Inc.). Statistical significance was set at p < 0.05. All reported p values were two-sided. Student’s t-test was used for comparison of marker expression between primary and recurrent tumors. To test for correlations between stem cell markers we used Pearson product-moment correlation coefficient. Kaplan–Meier analysis using log-rank statistics were used for comparing overall survival. Groups were divided by median expression values of the corresponding marker for their primary or recurrent tumor. Overall survival (OS) was defined as the time from the first tumor resection until death or last follow-up.

## Results

### Patients

Sixty paired glioma samples were collected from 30 patients treated at the department of neurosurgery from 07/2004 to 04/2017. Median age at diagnosis was 45.8 years. Median follow-up was 24 months. Most patients were diagnosed with World Health Organization (WHO) grade IV glioblastoma, one with grade II diffuse astrocytoma and one with grade III anaplastic astrocytoma. Their corresponding recurrent tumors progressed to grade III and IV glioma, respectively. Median duration from first two second surgery was 8.1 months. Patient and treatment characteristics are summarized in Table 1 and Additional file 1: Table S1.

### Staining for potential stem cell markers and osteopontin in paired tumor samples

**Fig. 1:**
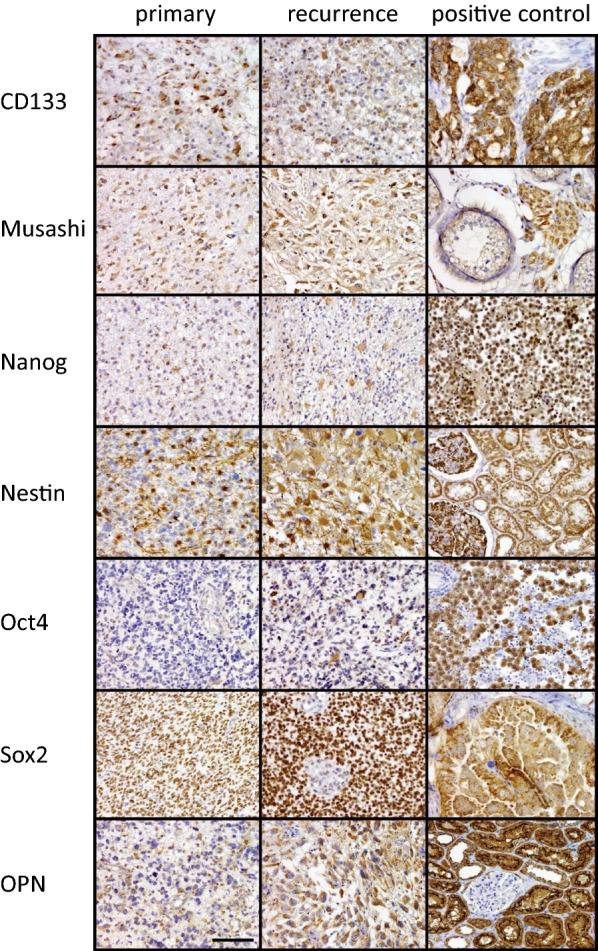
Representative IHC staining of glioblastoma specimens. Each row shows the expression of the corresponding stem cell marker in the primary and recurrent tumor. The right column shows the staining of the positive control tissue (CD133: colon cancer; Musashi: testis; Nanog, Oct4: seminoma; Nestin: kidney; Sox2 and OPN: ovarian cancer). Scale bar for all images: 100 µm

**Fig. 2:**
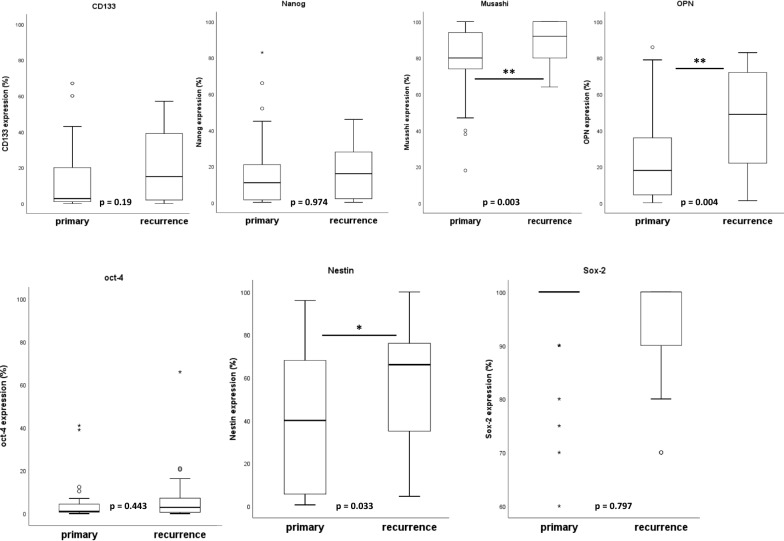
Levels of stem cell marker expression in primary and recurrent tumor. The relative number of positively stained glioma cells is shown as boxplot diagram for each marker. A significant increase in marker expression was seen for Musashi, Nestin and Osteopontin

Expression of the stem cell markers CD133, Musashi, Nanog, Nestin, Oct4 and Sox2 were analyzed on sections from primary and recurrent tumors by immunohistochemical staining (see Fig. 1). The staining of CD133 increased slightly from 13.4% in the primary to 19.3% in relapsed tumors (mean values, p = 0.19). Staining for the RNA binding protein Musashi was detected in cytoplasm and was significantly elevated by 12.2% in the recurrent tumors (77% versus 89.2%, p = 0.003). Nanog was found in nucleus to a small extent (0.2% versus 0.1%) as well as in cytoplasm with almost equal percentages in primary and recurrent tumors (17.3% versus 17.2%, p = 0.97). Nestin staining in cytoplasm clearly increased from 39.4% in primary tumor to 54.1% in relapses (p = 0.03). The transcription factor Oct4 was present in the nucleus in small amounts, both in the primary tumors and in recurrences (5% versus 7%, p = 0.44). The opposite is true for Sox2. In the primary tumors we found an expression rate of already 95%, which remained at high levels in relapses (95% versus 94.3%, p = 0.80). The amount of OPN expression increased significantly from primary to recurrent tumors (27% versus 46.1%, p = 0.004). Figure 2 shows the corresponding boxplot diagrams for each marker.

### Correlation between osteopontin and stem cell marker expression

We investigated a possible correlation between OPN and CSC marker expression. A significant correlation between OPN and CD133 (R = 0.68, p ˂ 0.01) and Nanog (R = 0.67, p ˂ 0.01) was seen in primary tumors. This association was still detectable in tumor recurrence between CD133 and OPN (R = 0.43, p ˂ 0.01) and Nanog (R = 0.62, p = 0.02).

Also, a significant correlation between CD133 and Nestin expression was seen in recurrence (R = 0.40; p = 0.03).

### Expression of stem cell markers according to IDH-1/2 status

**Fig. 3:**
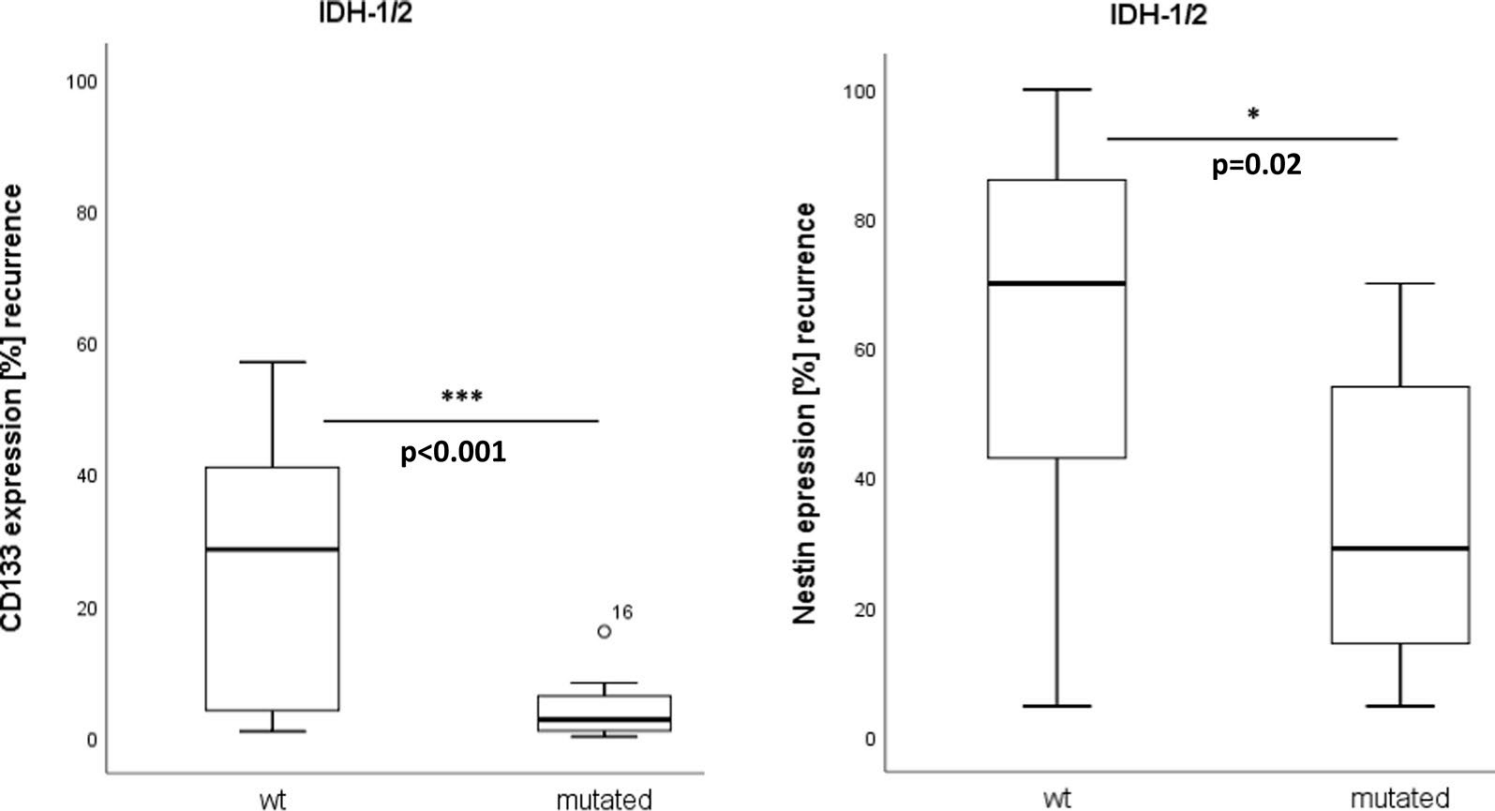
Stem cell marker expression according to IDH-1/2 mutation status. In recurrent tumors, expression of CD133 and Nestin is significantly increased for IDH-1/2 wild-type gliomas. The relative number of positively stained glioma cells is shown for CD133 and Nestin as boxplot diagrams

In our cohort, eight patients showed a mutated IDH-1/2 status and had significantly less expression of CD133 (4.4% vs 26.1%, p < 0.001) and Nestin (33.7% vs 62%, p = 0.02) in their corresponding recurrent tumors compared to patients with wild-type IDH-1/2 status. Figure 3 shows the corresponding boxplots.

### Co-localization between CD133 and Nestin

**Fig. 4:**
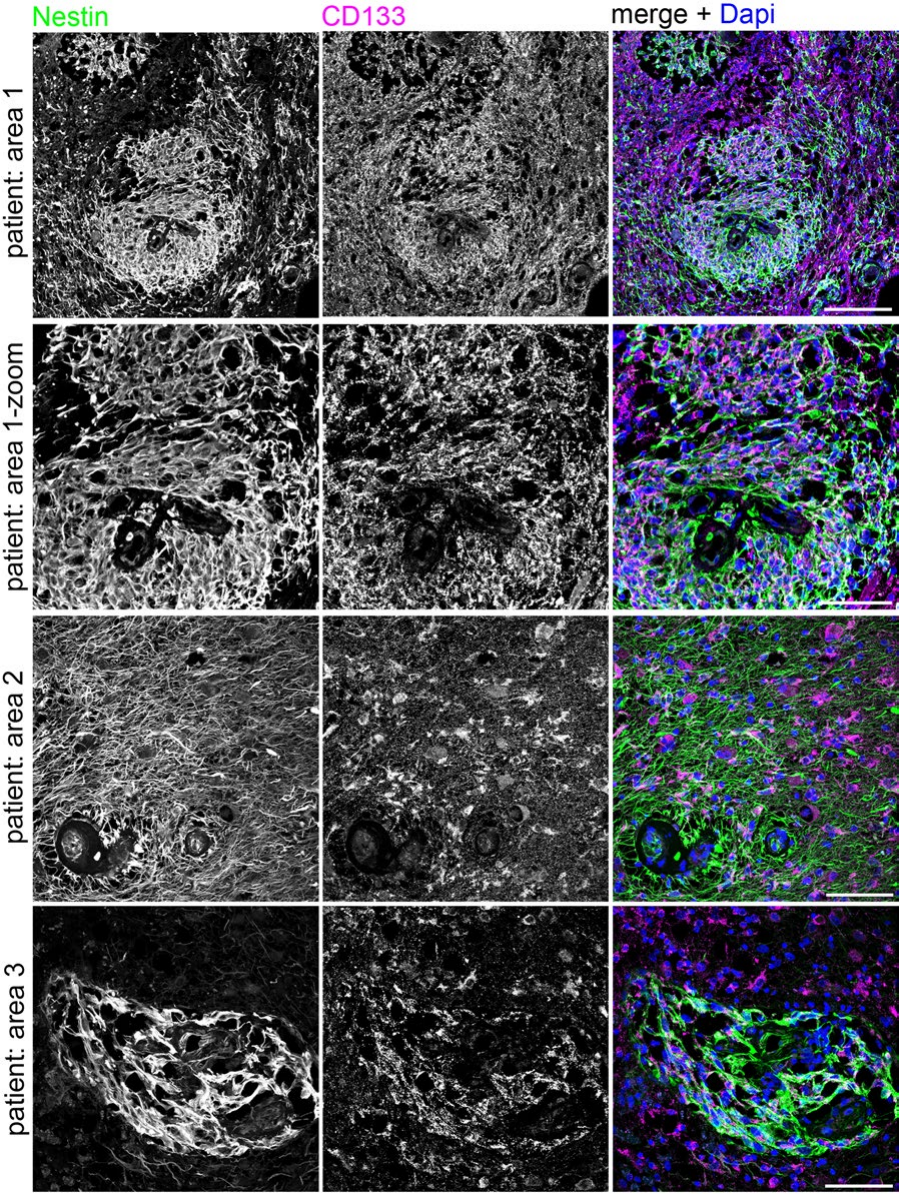
Nestin and CD133 immunoreactivity in glioblastoma. Nestin (green) and CD133 (magenta) immunofluorescence signals in a representative glioma section. DAPI was used as nuclear counter stain (blue). Yellow arrows in panel 3 and panel 4 point to Nestin + cells with a pronounced perinuclear CD133 signal. Shown are images acquired from different areas of the same tissue sample (recurrent glioma). Confocal images, maximum intensity projections, scale bars: panel 1: 150 µm; panel 2–4: 75 µm

After establishing simultaneous immunofluorescence staining for CD133 and Nestin we investigated if a co-localization of both markers could be detected within the same tumor cells. In a qualitative analysis from recurrent tumor samples using confocal 3D-microscopy, we clearly identified tumor areas where single tumor cells expressed both stem cell markers (see Fig. 4 and Additional file 2: Figure S1).

### Association of stem cell marker expression with overall survival

**Fig. 5:**
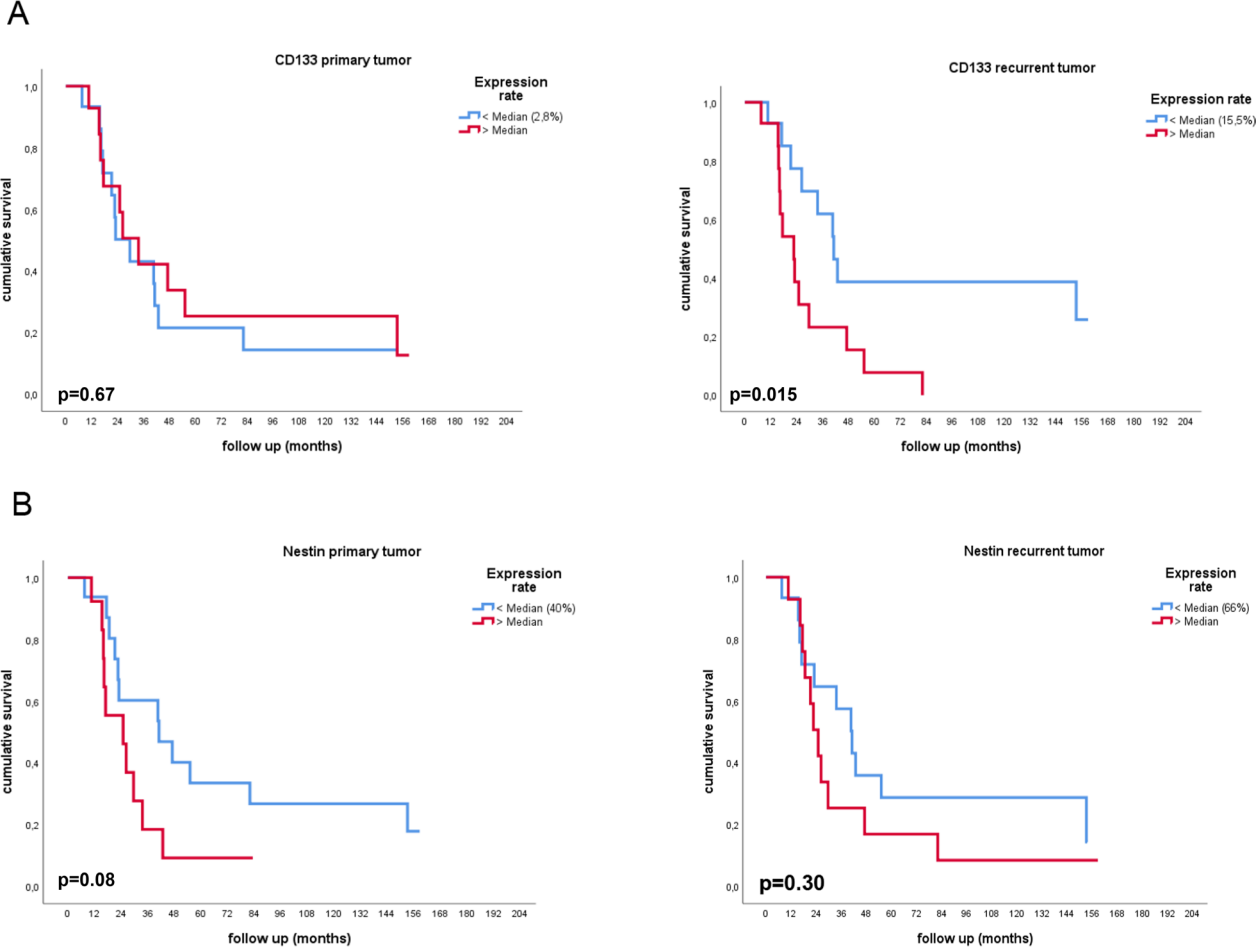
Overall survival shown by Kaplan–Meier curves for CD133 (**A**) and Nestin (**B**) in each primary and recurrent tumor. Curves are divided by the median expression rate of the corresponding marker

We investigated if stem cell marker expression was associated with overall survival. The groups were divided by the median amount of marker expression both in primary and recurrent tumors. We detected a significant association between CD133 expression and survival. In recurrent tumors a higher than median expression level (15.5%) correlated with a shorter median survival of 22.6 vs 41.1 months, p = 0.015 (see Fig. 5A). A similar observation was detected for Nestin expression in the primary tumors. Patients with a median expression of > 40% had a decreased survival time of 24.9 vs 41.1 months. The mean difference between both groups was 16.2 months (p = 0.08) (see Fig. 5B). For all other stem cell markers, we could not identify a correlation with overall survival (see Additional file 3: Figure S2). For Sox2 we did not perform survival analysis because the expression of this marker was highly abundant in almost all cells in primary and recurrent tumors (see Figs. 1, 2). Median OPN expression rate was 18% and 50% in primary tumors and recurrences, respectively. There was no significant association with overall survival when divided by the median expression rate (see Additional file 3: Figure S2).

### Influence of MGMT methylation, IDH-1/2 mutation and ATRX expression on survival

In our study, 13 of 30 patients (43%) showed a MGMT promotor methylation that has been proved to be a favorable prognostic factor irrespective of treatment. Median survival was 47.1 months in that subgroup compared to 22.6 months in patients with an unmethylated MGMT promotor, p = 0.036. Next, we examined the samples for IDH-1/2 (IDH1 R132H) status. Eight of 30 patients (27%) showed an IDH-1/2 mutation in association with a longer overall survival with 23 months vs 153.2 months, p = 0.001. Patients with IDH-1/2 mutation showed a methylated MGMT promotor in 6/8 (75%) and loss of nuclear ATRX expression in 7/8 (88%). In total we detected 9 patients (30%) with loss of ATRX function, which was associated with improved survival from 23 to 153.2 months, p = 0.02. Kaplan–Meier curves for overall survival are shown in Additional file 4: Figure S3.

## Discussion

Despite surgery and adjuvant radio-chemotherapy, patients with glioblastoma are condemned to consecutive recurrences. The recurrent tumors are at least as aggressive and resistant to therapy as the primary tumors and are thought to be initiated by persisting cancer stem cells, which are resistant to radio- and chemotherapy. Developing a therapy directed against these stem cells could be the “treatment of choice”. However, expression patterns of candidate stem cell markers between primary and recurrent tumors and their possible impact on survival have not been investigated thoroughly so far [37–40]. We identified a significant increase of Musashi and Nestin expression from primary to recurrent tumors in our cohort. Furthermore, our data showed a trend for reduced survival in patients with high levels (> 40%) of Nestin expression in their initial tumor. This finding is consistent with the meta-analysis by Lv et al. who showed that expression of Nestin increased with WHO grade and was associated with poor survival [41]. In regard to Musashi, we also detected high expression rates in most tumor cells (77%) already in the primary tumor, but could not see an association with overall survival. This is in part controversial to the work by Dahlrot et al. who investigated more than 200 glioma cases for Musashi expression. They demonstrated a worse prognosis for patients with high levels of Musashi expression in WHO grade III but not in grade IV glioma [42].

Regarding the expression of CD133, we could demonstrate reduced survival rates in patients with high CD133 levels in their recurrent tumor. Our results are consistent with the findings of Zeppernick et al. and the meta-analysis by Wu et al. [43, 44]. They confirmed the inverse association between the number of CD133 positive cells and survival, suggesting CD133 as a prognostic factor. Interestingly, we found a correlation between CD133 and Nestin expression levels in recurrent tumors (R = 0.40). Therefore, we investigated a possible co-localization of both markers within the tumor cells. With confocal laser scanning microscopy, we were able to detect clusters of tumor cells expressing both, CD133 and Nestin. In contrast, data from Dahlrot et al. showed conflicting results in regard to their prognostic impact when both markers were analyzed simultaneously [45]. Tamura et al. also detected an increase of CD133 in tumor recurrences after primary treatment [37]. This was mainly detected in de novo glioblastoma but not in secondary GB. In our study population eight patients had an IDH1 (R132H) mutation (indicating secondary GB), who showed lower levels of CD133 (4.4 vs 26.1%). Furthermore, we found a significant association between CD133 and OPN in primary and recurrent tumors, as well as a significant increase of OPN expression in recurrence. The group of Hira et al. detected in periarteriolar niches of high grade glioma patients residing CD133 + and Nestin + cells, which showed increased OPN expression [46]. Those hypoxic niches represent a special environment for CSCs, where they can proliferate and where the CSCs seem to be protected from radio- and chemotherapy. In these areas OPN seems to play an important role in promoting maintenance of stemness characteristics. Recently, we could show that OPN is overexpressed under hypoxic conditions in glioma cell lines [29] and other groups could demonstrate elevated OPN serum levels in high-grade glioma patients [28]. Furthermore, the meta-analysis by Zhao et al. clearly showed a correlation between OPN expression and prognosis [27]. In our study we observed a significant correlation between the expression levels of OPN and the transcription factor Nanog in primary and recurrent tumors. Recently, Wang et al. demonstrated that hypoxic conditions promote the high expression of Nanog in glioma, inducing formation of CSCs [47]. Due to all of these observations, we hypothesize that the simultaneous increase of Nanog and OPN expression may be related to the hypoxic milieu within the tumor microenvironment.

Oct4 is another transcription factor, which plays an important role for self-renewal and maintenance of pluripotency. It is expressed in human brain tumors and the degree of its expression positively correlates with tumor grade [48]. We could not observe an association between Oct4 expression and OS. This is in accordance with the findings of Petersen et al. suggesting no prognostic impact for Oct4 [49].

Additionally, we examined the influence of molecular markers for prognostic significance in malignant glioma, including MGMT promotor methylation status, IDH-1/2 mutation and loss of ATRX on overall survival. The results confirmed the expected survival benefit for patients with MGMT promotor methylation in combination with IDH-1/2 mutation and loss of ATRX. It is known that about 40–45% of glioblastoma patients show a methylated promotor region. In our study, 43% of the patients had a methylated MGMT promotor status, associated with a median survival of 23.1 months. MGMT methylation was only determined from untreated specimen because methylation status is largely consistent in primary and recurrent glioblastoma [50–52]. A mutation of the IDH-1/2 enzyme (R132H) was seen in 27% of the patients, which was associated with improved prognosis. In addition, we could show that recurrent tumors with wild-type IDH-1/2 status had elevated levels of Nestin and CD133. That fits together with the worse prognosis for patients with IDH-1/2 wild-type status. The association of CD133 and IDH-1/2 status was described before by Shibahara et al. but not for Nestin in patients with grade III glioma [53]. The exact molecular pathways of how CD133 and Nestin expression are linked to IDH-1/2 status, however, are still not fully understood.

This work has certainly some limitations, mainly by its retrospective nature and the small sample size. We must further assume a selection bias due to the limited availability of paired tumor samples only from patients who were amenable for secondary surgery. There are also difficulties in stem cell marker detection via IHC. This is especially true for CD133 staining since there are many post-translational modifications like glycosylation and different antibodies may recognize discrete epitopes on intra- or extracellular domains [54]. Comparability with the literature is further limited due to different staining and scoring methods.

To our knowledge this is the first study investigating multiple glioma stem cell markers together with osteopontin in samples from corresponding primary and recurrent gliomas.

In conclusion, most of the analyzed glioma stem cell markers showed an upregulation in their corresponding recurrent tumor. Patients with high rates of Nestin expression within the primary tumor tended for worse prognosis as did patients with an increased amount of CD133 positive tumor cells in recurrence. Furthermore, we observed that the worse prognosis in IDH-1/2 wild-type patients may be linked to an increased expression of CD133 and Nestin making these markers attractive candidates for further clinical investigations.

### Conclusions

The expression of Nestin, Musashi and OPN was significantly increased in recurrent glioblastoma. There was also a not significant increase for CD133 and Oct4. Especially, IDH-1/2 wild-type tumors showed an increased CD133 and Nestin upregulation. Both markers were co-localized within the same tumor cells and were associated with a worse prognosis. Our study warrants further stem cell marker directed treatment approaches, targeting CD133 and Nestin in particular.

## Supplementary Information

**Additional file 1: Table S1.** Detailed patient characteristics.

**Additional file 2: Figure S1.** Nestin (green) and CD133 (magenta) immunofluorescence signals in representative sections of the recurrent tumor from four different patients. DAPI was used as nuclear counter stain (blue). Confocal images, maximum intensity projections, scale bar: 150 µm.

**Additional file 3: Figure S2. **Overall survival shown by Kaplan-Meier curves for Musashi (**A**), Nanog (**B**), Oct4 (**C**) and Osteopontin (**D**) expressed in primary and recurrent tumor. Curves are divided by the median expression rate of the corresponding marker. None of the markers was significantly associated with overall survival.

**Additional file 4: Figure S3. **Overall survival shown by Kaplan-Meier curves for standard molecular markers. Survival was dichotomized by IDH-1/2 mutational status (**A**), MGMT promotor methylation status (**B**) and nuclear ATRX expression (**C**).

## Data Availability

The datasets used and/or analyzed during the current study are available from the corresponding author on reasonable request.

## Abbreviations

Oct4: Octamer-binding transcription factor 4
SOX2: Sex determining region Y-box 2
OPN: Osteopontin
CD133: Cluster of differentiation 133
IDH1/2: Isocitrate dehydrogenase 1/2
GB: Glioblastoma
CSCs: Cancer stem-like cells
ESCs: Embryonal stem cells
IHC: Immunohistochemistry
FFPE: Formalin fixed and paraffin embedded
H&E: Hematoxylin and eosin
GFAP: Glial fibrillary acidic protein
ATRX: Alpha-thalassemia/mental retardation syndrome X-linked
MGMT: O^6^‐Methylguanine DNA‐methyltransferase
DAB: 3, 3′-Diaminobenzidine tetrahydrochloride
TBS: Tris-buffered saline
DAPI: 4′, 6-Diamidino-2-phenylindole
OS: Overall survival
WHO: World Health Organization
