# Supplementary material for: Differences in stem cell marker and osteopontin expression in primary and recurrent glioblastoma
Source: Cancer Cell Int. 2022 Feb 19;22:87. doi: 10.1186/s12935-022-02510-4 (PMC8858483; doi:10.1186/s12935-022-02510-4)
Supplement: Supplementary file 1 — Additional file 1: Table S1. Detailed patient characteristics. [file 12935_2022_2510_MOESM1_ESM.docx]

**Table S1**

| **Patient / Gender** | **Age at diagnosis** | **WHO**  **Primary** | **WHO**  **Recurrence** | **MGMT** | **IDH-1/2** | **ATRX - Expression** | **follow up** | **Treatment**  **first line** | **Treatment**  **second line**  **(third, fourth, fifth line)** |
| --- | --- | --- | --- | --- | --- | --- | --- | --- | --- |
| 1 / ♀ | 39.7 | II | III | M | mut. | lost | 40.6 | S | S + RC |
| 2 / ♀ | 23.2 | III | IV | - | mut. | lost | 153.2 | S + RC | S + C, (S + R, C) |
| 3 / ♀ | 26.7 | IV | IV | - | - | retained | 10.6 | S + RC | S, (S, RC) |
| 4 / ♂ | 49.5 | IV | IV | M | mut. | retained | 105.4 | S + RC | S + C |
| 5 / ♀ | 69.4 | IV | IV | - | - | retained | 18.6 | S + RC | S |
| 6 / ♀ | 54.6 | IV | IV | - | - | retained | 41.1 | S + RC | S + C |
| 7 / ♀ | 59.1 | IV | IV | - | - | retained | 17.4 | S + RC | S + C |
| 8 / ♂ | 28.0 | IV | IV | - | mut. | lost | 158.7 | S + RC | S, (S) |
| 9 / ♀ | 39.9 | IV | IV | M | mut. | lost | 10.9 | S + R | S + C |
| 10 / ♂ | 45.8 | IV | IV | - | - | retained | 23.0 | S + RC | S + C |
| 11 / ♂ | 37.7 | IV | IV | - | - | retained | 21.2 | S + RC | S + C |
| 12 / ♀ | 27.4 | IV | IV | M | mut. | lost | 83.4 | S + RC | S + C |
| 13 / ♂ | 24.3 | IV | IV | M | mut. | lost | 153.6 | S + RC | S + RC, (S, S, C) |
| 14 / ♂ | 49.3 | IV | IV | M | - | retained | 16.0 | S + RC | S + RC |
| 15 / ♀ | 59.6 | IV | IV | M | - | retained | 12.5 | S + RC | S + C |
| 16 / ♂ | 34.9 | IV | IV | M | mut. | lost | 7.5 | S + RC | S + R |
| 17 / ♂ | 64.1 | IV | IV | - | - | retained | 17.0 | S + RC | S + C |
| 18 / ♀ | 41.3 | IV | IV | - | - | retained | 15.6 | S + RC | S |
| 19 / ♂ | 43.4 | IV | IV | M | - | retained | 82.1 | S + RC | S + RC |
| 20 / ♂ | 65.9 | IV | IV | - | - | retained | 24.9 | S + RC | S + RC |
| 21 / ♀ | 35.0 | IV | IV | - | - | retained | 16.3 | S+ RC | S + C |
| 22 / ♂ | 47.2 | IV | IV | - | - | retained | 29.6 | S + RC | S + R |
| 23 /♂ | 40.3 | IV | IV | - | - | lost | 22.6 | S + RC | S + C |
| 24 / ♀ | 65.4 | IV | IV | M | - | retained | 42.8 | S + RC | S + RC |
| 25 / ♂ | 42.4 | IV | IV | M | - | lost | 47.1 | S + RC | S + C |
| 26 / ♂ | 51.5 | IV | IV | - | - | retained | 26.3 | S | S + RC |
| 27 / ♂ | 49.1 | IV | IV | M | - | retained | 55.1 | S + RC | S + C, (S + C, R, C) |
| 28 / ♂ | 66.9 | IV | IV | - | - | retained | 15.4 | S + RC | S + C |
| 29 / ♀ | 45.8 | IV | IV | - | - | retained | 12.6 | S + RC+TTF | S + C + TTF |
| 30 / ♂ | 47.9 | IV | IV | M | - | retained | 33.6 | S + RC | S + C |

♀ female, ♂ male, MGMT methylation status of MGMT gene (M: methylated), IDH-1/2 Mutation status of IDH-1/2 gene (mut.: mutated), ATRX expression of nuclear ATRX, follow-up = time from 1^st^ surgery until date of last follow up, S = Surgery, R = Radiotherapy, C = Chemotherapy, RC = Radio-Chemotherapy, TTF = Tumor Treating Fields. Additional treatment beyond 2^nd^ line are within brackets and separated by comma.

**Supplementary Figure Captions**

**Figure S1.** Nestin (green) and CD133 (magenta) immunofluorescence signals in representative sections of the recurrent tumor from four different patients. DAPI was used as nuclear counter stain (blue). Confocal images, maximum intensity projections, scale bar: 150 µm.

**Figure S2.** Overall survival shown by Kaplan-Meier curves for Musashi (A), Nanog (B), Oct4 (C) and Osteopontin (D) expressed in primary and recurrent tumor. Curves are divided by the median expression rate of the corresponding marker. None of the markers was significantly associated with overall survival.

**Figure S3.** Overall survival shown by Kaplan-Meier curves for standard molecular markers. Survival was dichotomized by IDH-1/2 mutational status (A), MGMT promotor methylation status (B) and nuclear ATRX expression (C).
