# Supplementary figures and images for: Differences in stem cell marker and osteopontin expression in primary and recurrent glioblastoma
Source: Cancer Cell Int. 2022 Feb 19;22:87. doi: 10.1186/s12935-022-02510-4 (PMC8858483; doi:10.1186/s12935-022-02510-4)

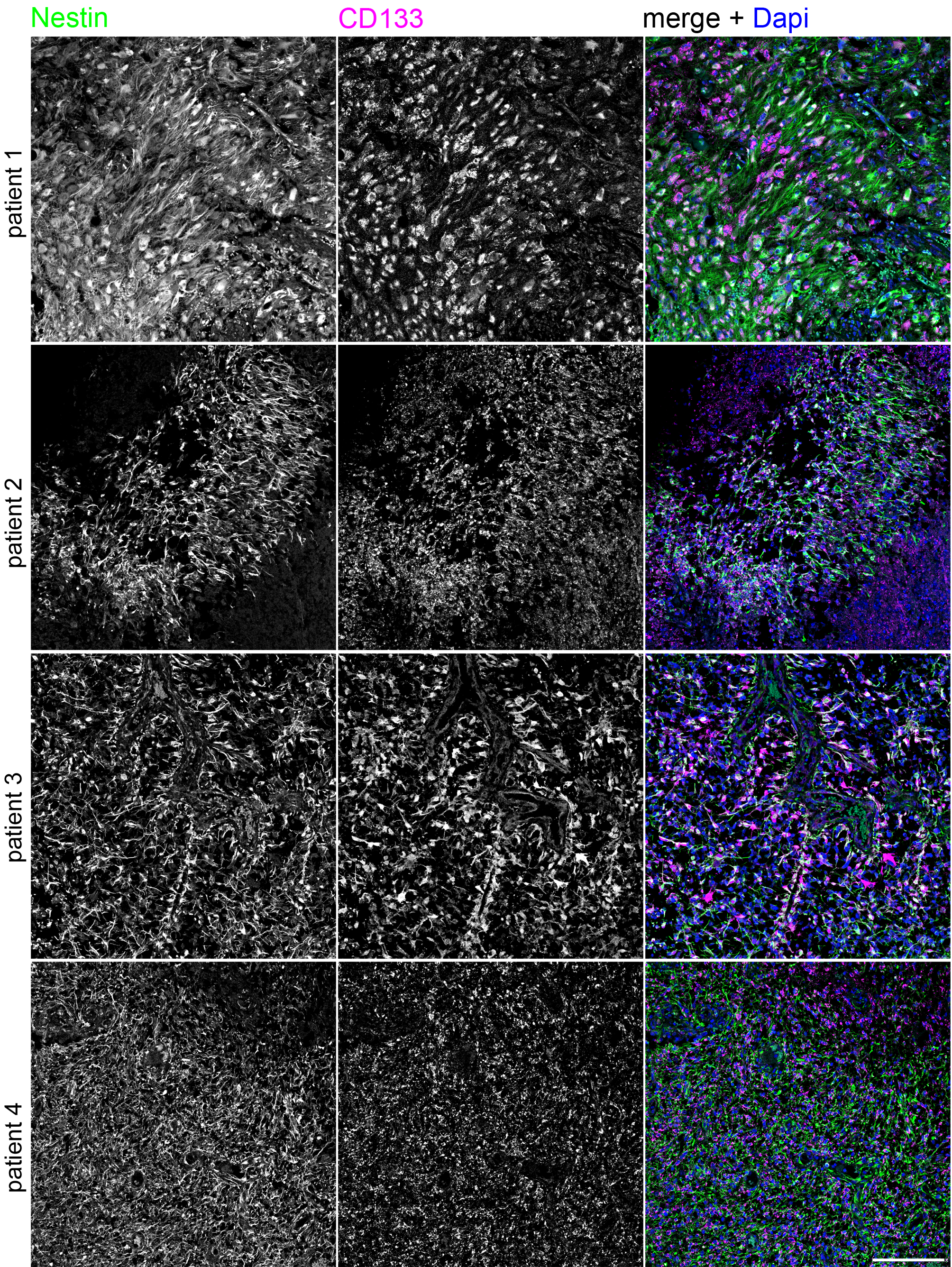

Supplement: Supplementary file 2 — Additional file 2: Figure S1. Nestin (green) and CD133 (magenta) immunofluorescence signals in representative sections of the recurrent tumor from four different patients. DAPI was used as nuclear counter stain (blue). Confocal images, maximum intensity projections, scale bar: 150 µm. [file 12935_2022_2510_MOESM2_ESM.tif]

## Slide 1
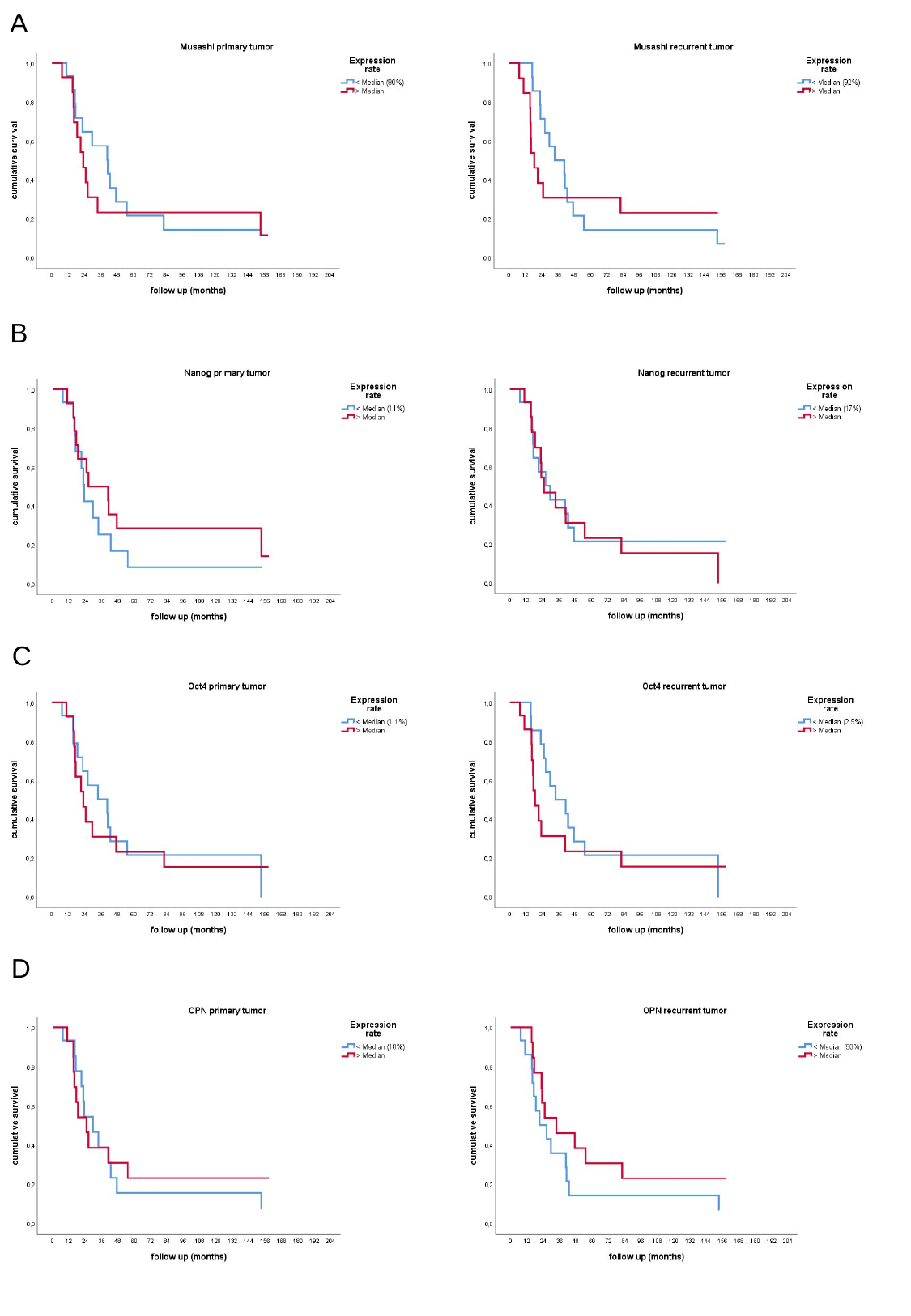

A
B
C
D

Supplement: Supplementary file 3 — Additional file 3: Figure S2. Overall survival shown by Kaplan-Meier curves for Musashi (A), Nanog (B), Oct4 (C) and Osteopontin (D) expressed in primary and recurrent tumor. Curves are divided by the median expression rate of the corresponding marker. None of the markers was significantly associated with overall survival. [file 12935_2022_2510_MOESM3_ESM.ppt]

## Slide 1
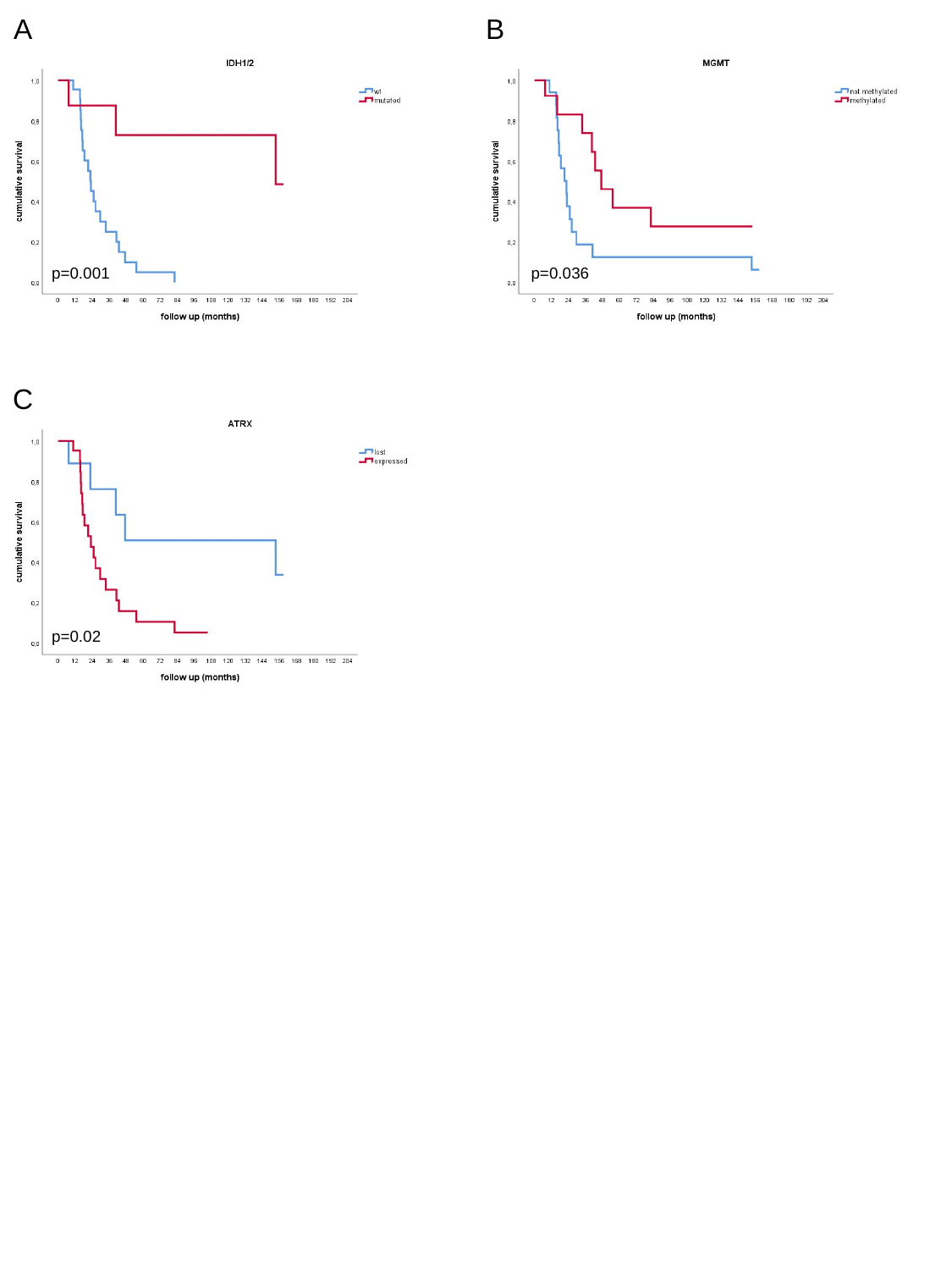

A
p=0.001
B
p=0.036
C
p=0.02

Supplement: Supplementary file 4 — Additional file 4: Figure S3. Overall survival shown by Kaplan-Meier curves for standard molecular markers. Survival was dichotomized by IDH-1/2 mutational status (A), MGMT promotor methylation status (B) and nuclear ATRX expression (C). [file 12935_2022_2510_MOESM4_ESM.ppt]
